# Suspected clinical toxoplasmosis in a 12-week-old puppy in Singapore

**DOI:** 10.1186/s12917-023-03674-5

**Published:** 2023-08-04

**Authors:** Audrey Chen, Max Boulay, Stacy Chong, Kelvin Ho, Amy Chan, Jasmine Ong, Charlene Judith Fernandez, Siow Foong Chang, Him Hoo Yap

**Affiliations:** 1 Centre for Animal Rehabilitation, Animal & Veterinary Service, Animal Management Centre, National Parks Board, 57 Sungei Tengah Road, 699013 Singapore, Singapore; 2 Westside Emergency, 41 Eng Kong Terrace, 599013 Singapore, Singapore; 3 Centre for Animal & Veterinary Sciences, Animal & Plant Health Centre, Animal & Veterinary Service, National Parks Board, 6 Perahu Road, 718827 Singapore, Singapore, Singapore; 4 Biorisk and Biosurveillance, Animal & Veterinary Service, National Parks Board, JEM Office Tower, 52 Jurong Gateway Road, 608550 Singapore, Singapore, Singapore; 5 Professional & Scientific Services, Animal & Veterinary Service, National Parks Board, JEM Office Tower, 52 Jurong Gateway Road, 608550 Singapore, Singapore, Singapore; 6 Animal & Veterinary Service, National Parks Board, Singapore Botanic Gardens, 1 Cluny Road, 259569 Singapore, Singapore

**Keywords:** Ascending paralysis, Sarcopenia, Toxoplasmosis, Free-roaming dog, Singapore

## Abstract

**Background:**

*Toxoplasma gondii* is traditionally known as a parasite of felids, with possible infection in intermediate hosts such as dogs and humans, and thus a disease of public health significance. Published data on the prevalence of toxoplasmosis in dogs and cats in Singapore is scanty, and this paper documents a suspect clinical case of toxoplasmosis in a free-roaming puppy trapped from an offshore island of Singapore.

**Case presentation:**

A 12-week-old puppy presented with hindlimb weakness and sarcopenia, with rapidly progressing ascending paralysis and respiratory distress, one week after trapping. Toxoplasmosis was suspected after indirect fluorescence antibody testing (IFAT) revealed anti-*T. gondii* antibodies. The puppy responded quickly to clindamycin treatment and was discharged from hospital after 10 days.

**Conclusion:**

While rare and undocumented, veterinary clinicians in Singapore are advised to also include toxoplasmosis infection as a differential diagnosis in dogs presenting with similar clinical signs. This is especially so for dogs which have access to the outdoors.

## Background

*Toxoplasma gondii* (which causes toxoplasmosis) is traditionally known as a parasite of felids, which are recognized as the only known definitive hosts. Humans and a wide range of animals, including dogs, can also become infected as intermediate hosts. There are numerous records on the prevalence of toxoplasmosis in dogs worldwide [1, 2]. A recent review found that the seroprevalence in dogs varied greatly between regions [3]. In this review, risk factors such as age, sex, and housing were reported as positive associations [3]. Primary infection with *Toxoplasma* in dogs is thought to be rare, and most reported cases are in dogs with co-infections or known immunosuppression [3].

Neurological signs, including paralysis, have been reported in dogs infected with *Toxoplasma gondii* [4–8]. Many of the dogs documented in these reports were puppies or had concurrent infections, such as canine distemper [4, 5, 7].

At the time of this report, there is no known published evidence of toxoplasmosis in dogs in Singapore, to the knowledge of the authors. However, due to the presence of significant numbers of free-roaming cats in the community, the risk of exposure of *T. gondii* oocysts in cat faeces to dogs is not insignificant. Rats have also been suggested as a possible reservoir of infection and transmission to dogs [9, 10]. Transplacental infection of toxoplasmosis in dogs has been reported to have occurred naturally in one case in Australia [11], and it has also been documented in experimentally inoculated animals [12].

## Case presentation

Two free-roaming dogs (FRD), one male (Dog 1) and one female (Dog 2), likely to be littermates and estimated to be about 12-weeks of age, were trapped on an island off Singapore and sent to the Animal Management Centre (AMC)^1^. At triage, both puppies were observed to be quiet and alert, and no abnormalities were detected on physical examination. Both puppies received the first dose of core canine vaccine (Boehringer Ingelheim Recombitek© C6/CV), a topical ectoparasiticide containing fipronil and (S)-methoprene (Frontline© Plus), and oral treatment containing pyrantel, oxantel and praziquantel (Ilium© Pyraquantal) for intestinal parasites. As part of an ongoing national biosurveillance programme for FRDs, plain and EDTA blood samples were obtained from both dogs to screen for *Leptospira* sp. (PCR), *Leishmania* sp. (SNAP Leishmania; IDEXX), *Dirofilaria immitis*, *Borrelia burgdorferi*, *Anaplasma* sp. and *Ehrlichia* sp. (SNAP 4Dx Plus; IDEXX). None of the above pathogens were detected in the two dogs. Blood parasites (e.g., *Babesia* sp.) were also not observed on the peripheral blood films of the two dogs. Both dogs were fed a commercial diet consisting of mixed dry kibble and canned wet food, with no access to uncooked animal product.

One week after admission into AMC, the puppies developed mild diarrhoea (Purina© faecal score 5–6). The clinical signs resolved within 3 days following treatment with oral crospovidone (100 mg/kg, BID). The following week, a small non-pruritic focal area of alopecia was noticed along the dorsum of Dog 1. Woods lamp examination of the two puppies was negative and dermatophyte culture (KRUUSE© Dermatophyte test) of hair plucking from around the alopecic area of Dog 1 was performed. Both puppies were given oral afoxolaner (Nexgard©) to empirically treat for potential demodicosis and daily application of a quaternary-ammonium compound based antiseptic ointment on the alopecic area (F10© Germicidal Barrier Ointment) was initiated. The two pups were shifted to a new kennel for isolation on a precautionary basis. The dermatophyte culture was negative after 10 days, and both dogs were given the second dose of core canine vaccine (about three weeks after the first dose). Both dogs were adopted into their new homes a few days later (3 weeks after intake).

**Fig. 1:**
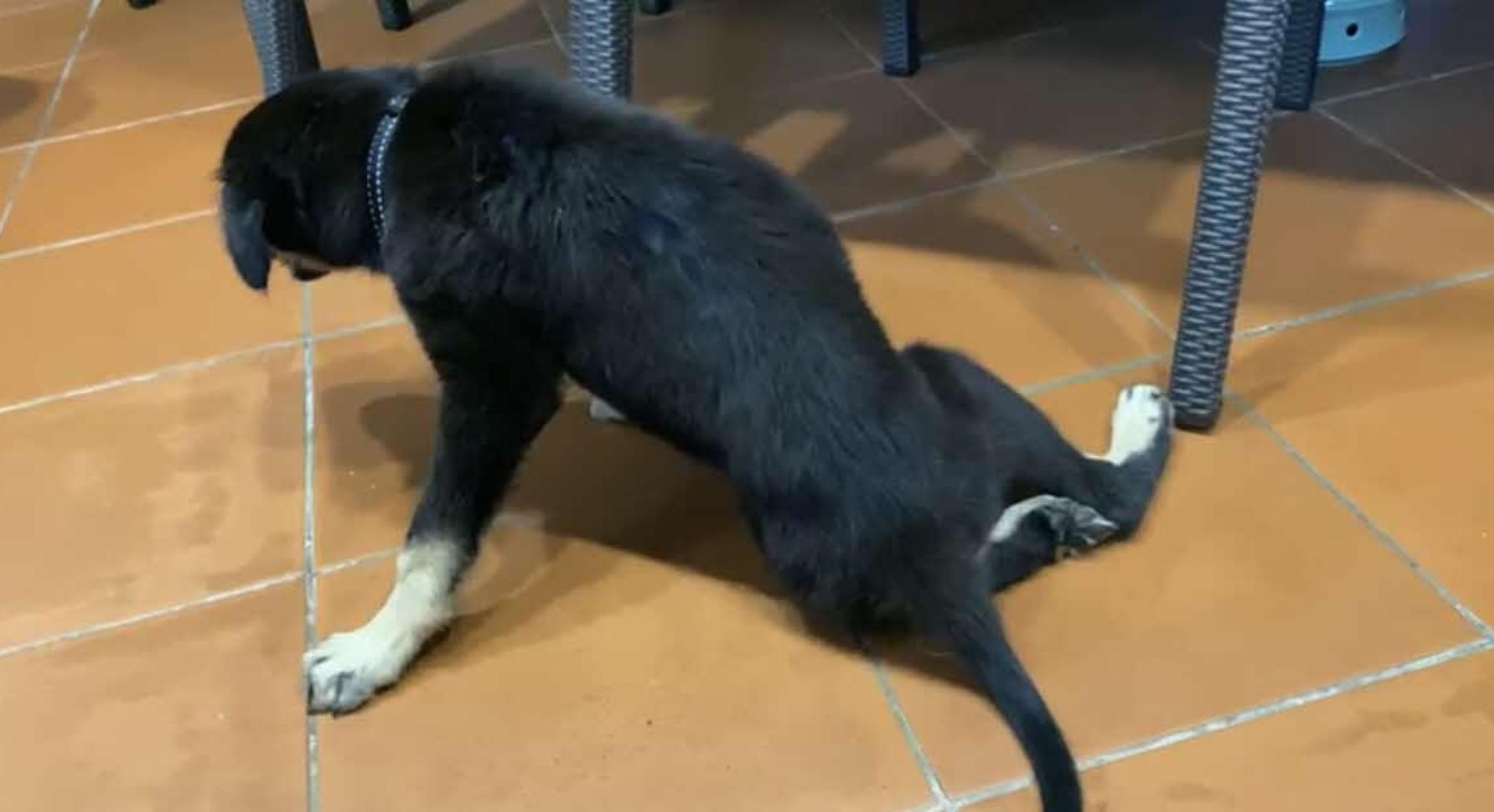
Hindlimb paresis in Dog 1

One week after adoption, the owner reported that the male puppy (Dog 1) was inappetent, dull and constipated. It also appeared to have progressive paresis in the hind legs (Fig. 1), and an occurrence of falling off a low chair, after onset of the paresis. The female puppy (Dog 2) was clinically healthy throughout. Dog 1 was examined at a veterinary clinic where slow general proprioception, bilateral sarcopenia in the hindlimbs, and mild lameness in the left hindlimb were observed by the attending veterinarian. Spine and hip radiographs were normal. A complete blood count showed mild non-regenerative anaemia (haematocrit 0.286 L/L; reference interval (RI) 0.373–0.617 L/L) that was microcytic (MCV 59.0 fL; RI 61.6–73.5fL) and non-regenerative (reticulocytes 75.2 K/µL; RI 10–110 K/µL), mild monocytosis (1.62 × 10^9^/L; RI 0.16–1.12 × 10^9^/L) and biochemistry results showed a mild elevation of globulins 4.0 g/dL (RI 2.3–3.8 g/dL). Screening for vector-borne diseases (SNAP 4Dx Plus; IDEXX) was repeated by the attending veterinarian, revealing that the dog was seropositive for *B. burgdorferi*. Subsequent PCR performed by the Centre for Animal and Veterinary Sciences (CAVS) did not detect *B. burgdorferi* DNA in the blood. The dog was empirically started on oral doxycycline (5 mg/kg BID), tramadol (2.5 mg/kg BID) and iron supplements. Differential diagnoses include spinal trauma due to the fall or a congenital orthopedic condition.

As the neurological signs continued to deteriorate, Dog 1 was referred to an emergency veterinary hospital after one day and was noted to be tachycardic (170 bpm), tachypnoeic with an increased effort and short shallow breaths, and pyrexic (39.5 °C). Neurological examination indicated moderate tetraparesis with the right limbs affected worse than the left, inability to support head or weight on limbs, and reduced patella and withdrawal reflexes. Cranial nerve assessment was normal with no evidence of spinal pain. Thoracic radiographs indicated a mild generalised bronchial pattern and a small radio-dense circular structure in the cranial abdomen, suspected within the stomach.

Haematology revealed anaemia (haematocrit 0.285 L/L; reference interval (RI) 0.373–0.617 L/L) that was microcytic (MCV 58.3 fL; RI 61.6–73.5 fL) and non-regenerative (reticulocytes 65 K/µL; RI 10–110 K/µL), mild monocytosis (1.34 × 10^9^/L; RI 0.16–1.12 × 10^9^/L), mild eosinopenia (0.05 × 10^9^/L; RI 0.06–1.23 × 10^9^/L), and moderate thrombocytopenia (42 × 10^9^/L; RI 148–484 × 10^9^/L). Biochemistry results were within normal clinical ranges, including creatine kinase and ionised calcium. SpO_2_ (96%), venous blood-gas, blood pH, PaCO_2_ and electrolytes were also within normal range.

The dog was initially managed with maropitant (1 mg/kg IV q24) and metoclopramide (0.33 mg/kg IV q8) to limit possible regurgitation; aminophylline (3 mg/kg IV q8); oxygen supplementation (200ml/kg/min via nasal oxygen tube) for respiratory support; and Hartmanns for fluid therapy.

The following morning, the dog was anaesthetised with methadone (0.2 mg/kg IV) as pre-medication and alfaxalone (1 mg/kg IV) as induction agent, intubated and maintained on oxygen and isoflurane. A cerebellomedullary cistern site puncture was attempted but no cerebrospinal fluid could be collected. The owner later reported that the dog had access to cycad trees and had been seen chewing on seeds within the last week. A gastroscopy was performed to investigate stomach contents, which only consisted of food material (kibbles, pumpkin) and some sand. Repeat abdominal radiography confirmed absence of the previously noted circular structure.

Further blood screening for acetylcholine receptor antibody were normal (0.13 nmol/L, RI < 0.6 nmol/L indicates normal serum titer) and *Neospora caninum* antibody were tested negative by IFAT at an external laboratory (IDEXX). Serological screening for *Toxoplasma gondii* via IFAT was performed at CAVS. Two-fold serial dilutions of serum, starting at 1:16, were prepared using serum diluting buffer. 20 µl of each serum dilution was added to a well on a substrate slide coated with *T. gondii* tachyzoites of RH strain (VMRD Inc, USA), and incubated for 30 min at 37°C in a humid chamber. The slide was then rinsed with Fluorescent Antibody (FA) rinse buffer and washed twice on an orbital shaker for 5 min and blotted dry. After which, 20 µl of anti-canine Immunoglobulin G (IgG) or Immunoglobulin M (IgM) antibody was applied to each well and subjected to the same incubation and wash steps. The slide was then examined using a fluorescence microscope (Olympus BX41, Japan) at 200x to 400x magnification. This revealed endpoint titres of 1:512 for (IgG) and 1:64 for (IgM) in the dog. With a working diagnosis of toxoplasmosis, the dog was started on oral clindamycin (20 mg/kg IV q12). Eye lubrication and regular limb physiotherapy were instituted as general nursing care.

Two days after treatment for suspected toxoplasmosis was initiated, the animal’s condition began to improve, with appetite returning, and it was able to be weaned off oxygen support by day-4 from admission to the emergency hospital. Due to the lack of ambulation, the dog had developed firm stools and constipation which were managed well with lactulose (0.4ml/kg PO q12). Head control improved by day-5, and the dog was standing supported by day-7. Dog 1 was released on day-10 from the hospital for ongoing home care with a 28-day course of clindamycin (15 mg/kg PO q12).

**Fig. 2:**
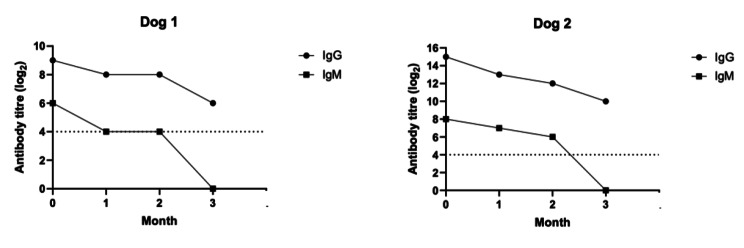
*Toxoplasma gondii* IgG and IgM antibody titres over a period of 3 months. The IFAT cut-off value of 1:16 is indicated by dashed lines. (A) IgG levels of Dog 1 had dropped three-log to a titre of 1:64, (B) while IgG levels for Dog 2 decreased five-log to a titre of 1:1024. For both dogs, IgM titres dropped below the cut-off value at the 3-month mark

Although Dog 2 was clinically healthy throughout this period, the attending veterinarian put it on a prophylactic 2-week course of clindamycin (15 mg/kg PO q12). A Serum sample was also collected from Dog 2 and screened similarly using IFAT. Though Dog 2 was non-clinical, its test indicated higher antibody titres of 1:32,678 for IgG and 1:256 for IgM. Thereafter, serum samples from both dogs were subjected to monthly IFAT to monitor the antibody titres over time. A consistent decrease in IgG and IgM levels was observed in both dogs every month; except for Dog 1 at the 2-month mark, where IgG and IgM titres remained stable. By the 3-month mark, IgM titres of both dogs were no longer detectable at the cut-off of 1:16 and IgG titres had dropped at least three-log. The antibody titres of both dogs over a three-month period are shown in Fig. 2.

By the 3-month mark, Dog 1’s neurological signs had fully resolved. A biopsy was taken from the *biceps femoris* muscle of Dog 1 when he was anaesthetised for routine castration. No *T. gondii* DNA was detected in the muscle sample via PCR.

### Further investigation in free-roaming dog population

As an extension of the national biosurveillance programme for free-roaming dogs, serum from free-roaming dogs (FRD) that were trapped from the same offshore island and admitted into AMC was opportunistically obtained and screened for IgG and IgM antibodies against *T. gondii*. From January 2021 to February 2022, 20 dogs were screened and one dog (5%) was seropositive (IFAT endpoint titre IgG 1:256 and IgM 1:258). The seropositive dog did not display any clinical signs suggestive of active infection with *T. gondii*.

## Discussion and conclusions

This is the first presumptive case of primary clinical canine toxoplasmosis documented in Singapore, to the best knowledge of the authors. Despite not detecting the presence of parasites in muscles, the clinical case was diagnosed with a combination of consistent clinical signs (ascending paresis and paralysis); serological screening for *T. gondii* and *N. caninum* (which ruled out the latter); ruling out of other factors including Lyme disease, trauma and cycad poisoning, and response to treatment with clindamycin.

Lyme disease, trauma and cycad poisoning were important differentials which could have contributed to the acute clinical presentation. Lyme disease was ruled out as test kit and laboratory PCR tests were negative. Lyme disease has also not been reported in dogs in Singapore and Singapore is not known to be endemic for the disease.

Trauma from the puppy’s fall from a low height was unlikely the causative factor in the absence of any radiological findings and unrelated to the rapid clinical progression to respiratory failure. From the history, it was more likely to have been secondary to the paresis and general weakness that the puppy was already experiencing.

Cycad poisoning from consumption of cycad seeds similarly was ruled out due to absence of clinical signs such as vomiting, diarrhoea and hepatic involvement.

In this case report, the young age of the puppy at the onset of clinical signs, as well as the high IgM antibody titres in both dogs, suggested that the dogs were infected around the same time. The IgM antibody titres subsequently dropped to undetectable levels after three months from the onset of clinical signs. Based on the kinetic profile of the IgM and IgG antibodies [13], it is postulated that the *T. gondii* infection is likely acute and probably occurred shortly before the onset of clinical signs. However, there were no pre-clinical serum samples to confirm this. It should also be noted that the interpretation of the IgG and IgM antibody titres is complicated by the paucity of studies between antibody titres, development of clinical signs and the chronicity of toxoplasmosis.

Given the anecdotal absence of cats in the area where the puppies were trapped, the involvement of other sources of infection such as wild felids could not be ruled out. Based on limited studies, the likelihood of detecting the presence of antibodies against *T. gondii* increases with age, due to the increased likelihood of exposure to oocysts disseminated into the environment from cat faeces. Due to their young age, the likelihood of concurrent exposure of both dogs to *T. gondii* oocysts from the environment was relatively low. A study by Arantes et al. [14] showed that toxoplasmosis could be sexually transmitted but it is worth highlighting that all the offspring from female dogs (n = 4) in that study that were artificially inseminated with semen from infected dogs did not survive past 18 days.

Most documented cases of primary clinical toxoplasmosis were either in puppies or were associated with immunosuppression [4, 5, 7]. In particular, infection with canine distemper virus has been highlighted to lower resistance to pre-existing *T. gondii* infection, and even vaccination with modified live canine distemper virus could aggravate the infection [15]. While there was no clear indication of an immunosuppressive condition in Dog 1, including the cause of its alopecia, it might be possible that the core canine vaccination, compounded by stress from the new shelter and home environment, could have predisposed to clinical progression in the dog. Dog 2, while asymptomatic throughout, was noted to have had higher IgM and IgG antibody titres than Dog 1. This stronger immune response could have explained the absence of clinical signs in Dog 2, and supports our postulate that the clinical signs were due to *T. gondii* infection.

While there is one limited study on the prevalence of toxoplasmosis in cats in Singapore which documented the prevalence to be around 5% [16], there is no information on the disease prevalence in dogs. While dogs are not the definitive host of *T. gondii*, infection could still be of public health relevance as humans could be exposed when in contact with dogs that have encountered contaminated cat faeces via the dogs’ body surfaces, mouth, or feet. This is especially so in Singapore’s context, where free-roaming cats are relatively common. The epidemiology of the disease in dogs, both on mainland and offshore islands of Singapore, hence, warrants further investigation.

### Recommendation

Although primary clinical toxoplasmosis is considered rare, this case report indicates the clinical importance of *T. gondii* infection in dogs (particularly those with outdoor access) and informs of the need for clinicians to include *T. gondii* infection in the list of differentials for dogs presenting with ascending paralysis and muscle wasting.

## Data Availability

All data generated or analysed during this study are included in this published article.

## Footnotes

1 Animal Management Centre (AMC) is an animal shelter operated by the Animal & Veterinary Service, a cluster under the National Parks Board of Singapore.
